# Supplementary material for: Centrality in the host–pathogen interactome is associated with pathogen fitness during infection
Source: Nat Commun. 2017 Jan 16;8:14092. doi: 10.1038/ncomms14092 (PMC5241799; doi:10.1038/ncomms14092)
Supplement: Supplementary Information — Supplementary figures, supplementary methods and supplementary references. [file ncomms14092-s1.pdf]

Supplementary Figures

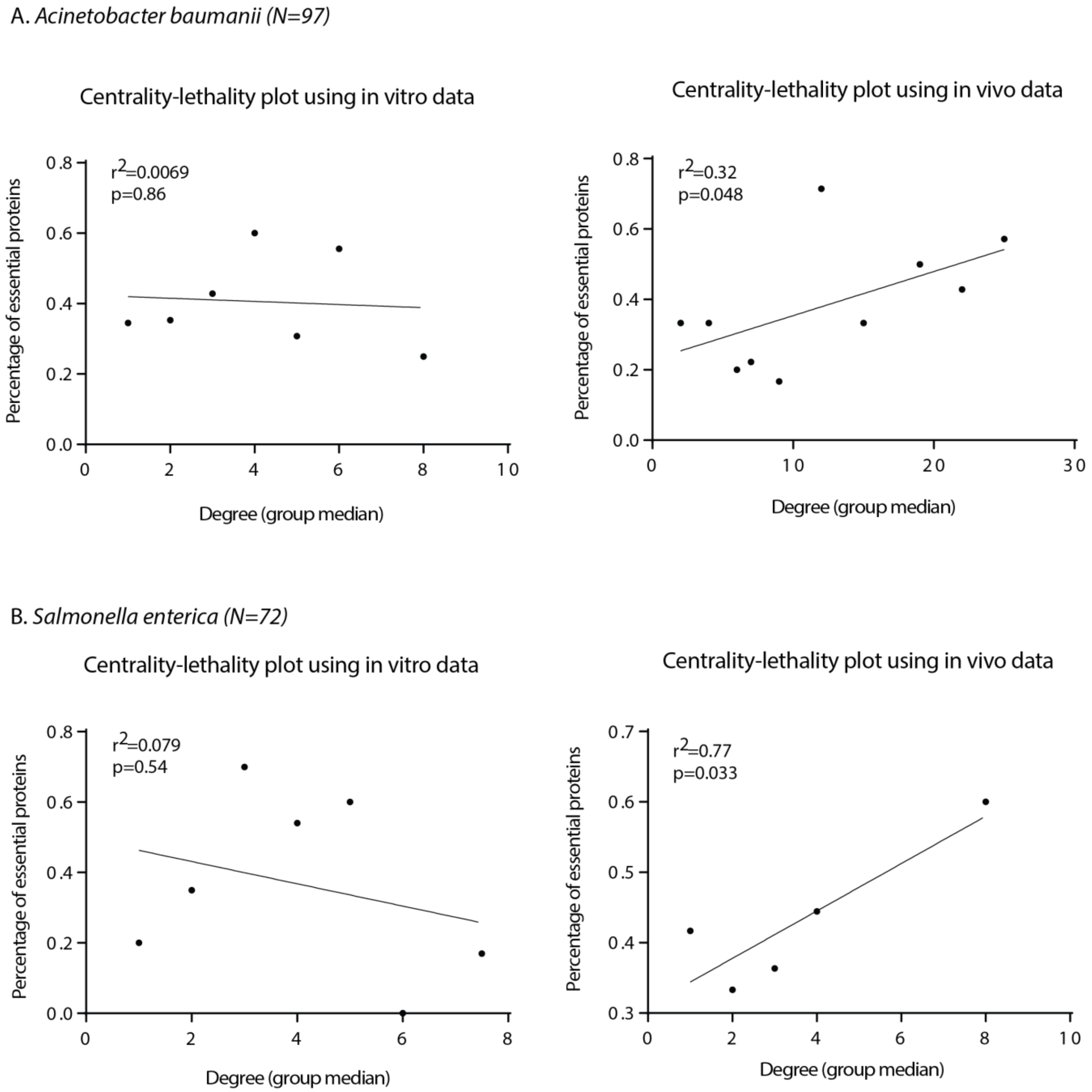

Supplementary Figure 1. *Analysis of the centrality-lethality rule in Acinetobacter Baumanii and Salmonella enterica.* The percentage of essential proteins for *A. baumanii* (A) and *S. enterica* (B) infection was plotted against its degree in the host (left) and the host-pathogen interactome (right). The total number of observations (*n*) is included in each graph (n=97 for the *H. sapiens-A. baumanii* interactome and n=72 for the *H. sapiens-S. enterica* interactome).

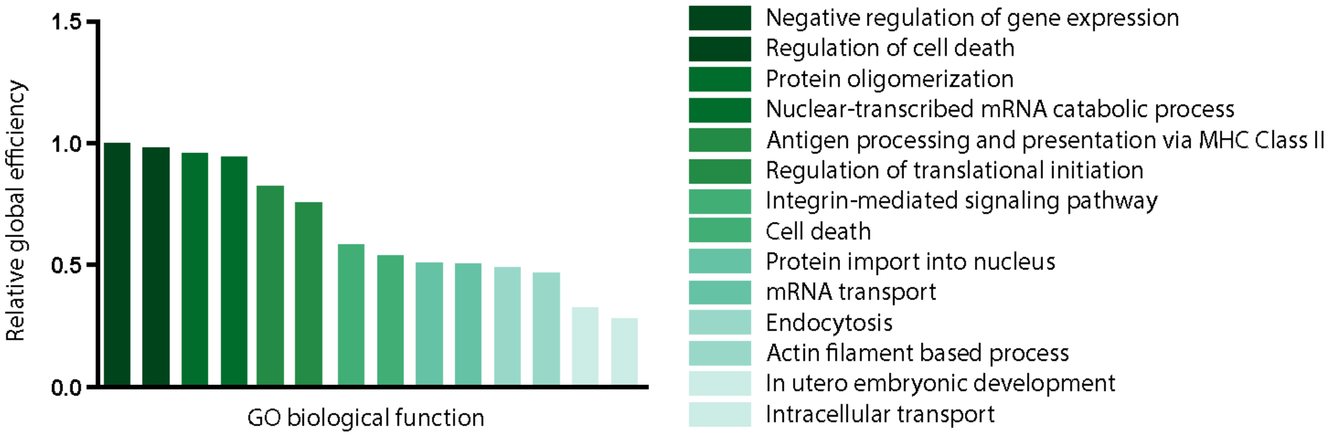

Supplementary Figure 2. *Change in relative global efficiency after pathogen-directed attach measured for all host networks targeted by Y. pestis*. Significant biological functions were obtained using David as described in the Methods section (adjusted p-value < 0.05) and interaction networks for each function were built using String. The relative global efficiency measured after a simulated pathogen-directed attack on each network is plotted.

Supplementary Methods

Analysis of potential biases in the definition of the centrality-lethality rule

Y. pestis-H. sapiens interactome was defined using a random yeast two-hybrid assay (Y2H) previously published by Dyer et al [1]. In Y2H high-throughput techniques, three biases can occur that can contribute to mask the results:

a. *Experimental design bias*: In contrast to the classic yeast two-hybrid (Y2H) screening, the dataset used in this study was obtained by random Y2H meaning that no selection was made prior to the experiment about which proteins would be analysed. The proteins contained in the dataset used in our study were randomly picked after yeast mating and were never subjected to previous selection.

b. *Small-scale dataset bias*: The dataset used in our study has 4.059 PPIs and comes from the inspection of >500.000 diploid yeast cells, i.e. screened protein-protein interactions. Hence, in our opinion, the dataset should not be considered as a small-scale study. In any case, to reinforce the validity of our results, we have conducted bootstrapping analyses to provide a confidence interval in all r^2 values in **Fig. 1**. The results obtained further support our claim that centrality-lethality rule only holds when the host-pathogen interactome is considered.

c. *Technical bias*: All datasets have technical bias, which is inherent to any experimental analysis. Such bias cannot be removed but can be controlled by comparing independent studies. Therefore, in order to strengthen our results we have tested the correlation between the dataset used and the data published by Yang et al. [2] where the authors studied the protein-protein interaction network of *Y. pestis* virulence factors. We observed that the shared coverage between the two datasets was very low (as also mentioned by Yang et al. in their paper [2]) though we could identify 12 proteins with information available in both datasets. Using this limited data, we asked whether proteins with highest impact on fitness have also a higher connectivity in Yang's dataset. Consistently with our previous results, we observed that proteins that were most essential in *Y. pestis* to infect the host had indeed a higher degree in the host-pathogen interactome (See **Supplementary Fig. 3**, p=0.039).

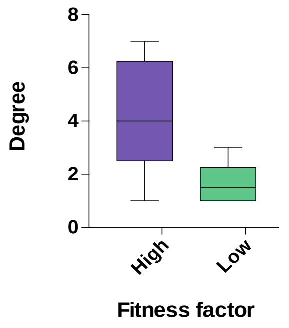

Supplementary Figure 3. Average degree for proteins classified by fitness factor in the dataset of *Yang et al. The number of observations was 6 in both groups.*

In order to further study how technical biases in the interactome could affect our conclusions, we simulated random perturbation assays in the degree measurements and repeated the analysis. The noise was generated using the jitter function in R and the amount of noise (n) can be described by $n = f\frac{d}{5}$, where d is the smallest difference between values and f a factor defined by the user. We observed that, even for f=25 the conclusions remained sound and r^2 values for the centrality-lethality rule *in vitro* and *in vivo* where 0.82±0.02 and 0.78±0.02, respectively.

Supplementary references:

1. Dyer MD, et al. The human-bacterial pathogen protein interaction networks of Bacillus anthracis, Francisella tularensis, and Yersinia pestis. *PloS one* 5, e12089 (2010).

2. Yang H, et al. Insight into bacterial virulence mechanisms against host immune response via the Yersinia pestis-human protein-protein interaction network. *Infection and immunity* 79, 4413-4424 (2011).
